# Supplementary material for: POT1a deficiency in mesenchymal niches perturbs B-lymphopoiesis
Source: Commun Biol. 2023 Sep 29;6:996. doi: 10.1038/s42003-023-05374-0 (PMC10541440; doi:10.1038/s42003-023-05374-0)
Supplement: Supplementary file 2 — Supplemental material [file 42003_2023_5374_MOESM2_ESM.pdf]

Supplementary Figures and Table

Title: POT1a deficiency in mesenchymal niches perturbs B-lymphopoiesis

Kentaro Nakashima[1,2] Yuya Kunisaki[1,3,4*], Kentaro Hosokawa[1], Kazuhito Gotoh[5], Hisayuki Yao[1], Ryosuke Yuta[1], Yuichiro Semba[3,4], Jumpei Nogami[3,4], Yoshikane Kikushige[3,4], Patrick S Stumpf[6], Ben D MacArthur[7,8,9], Dongchon Kang[5], Koichi Akashi[4], Shouichi Ohga[2], Fumio Arai[1*]

Corresponding to Yuya Kunisaki (kunisaki.yuya.519@cancer.med.kyushu-u.ac.jp) and Fumio Arai (arai.fumio.603@m.kyushu-u.ac.jp)

Lead contact: Fumio Arai (arai.fumio.603@med.kyushu-u.ac.jp)

Supplementary Figure 1

Supplementary Figure 2

Supplementary Figure 3

Supplementary Figure 4

Supplementary Figure 5

Supplementary Table 1

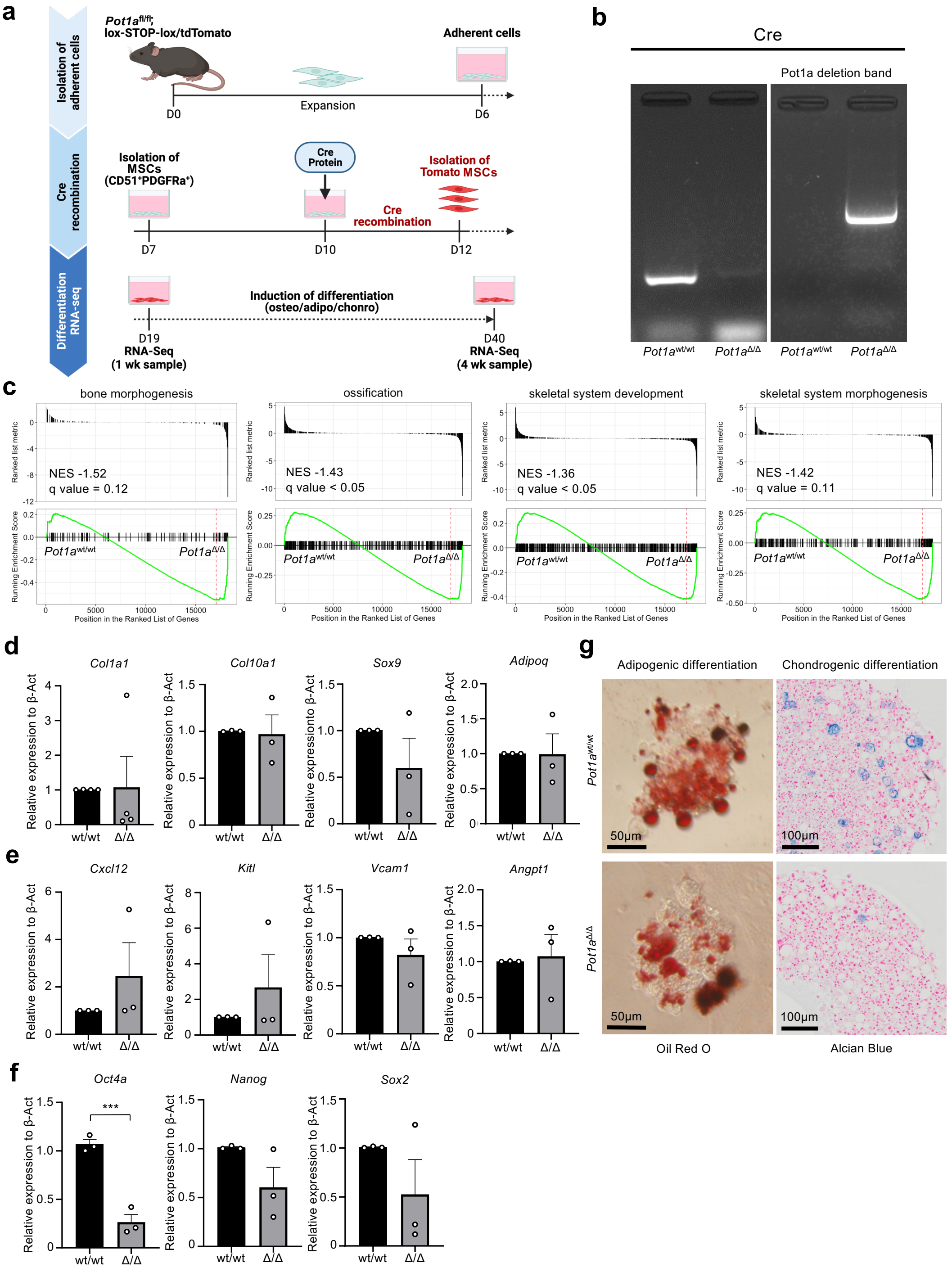
a
Isolation of adherent cells
Cre recombination
Differentiation RNA-seq
Pot1a fl/fl; lox-STOP-lox/tdTomato
Adherent cells
Expansion
D0
D6
Isolation of MSCs (CD51+PDGFRa+)
Cre Protein
Isolation of Tomato MSCs
Cre recombination
D7
D10
D12
Induction of differentiation (osteo/adipo/chonro)
D19 RNA-Seq (1 wk sample)
D40 RNA-Seq (4 wk sample)
b
Cre
Pot1a deletion band
Pot1a wt/wt
Pot1a Δ/Δ
c
bone morphogenesis
NES -1.52
q value = 0.12
ossification
NES -1.43
q value < 0.05
skeletal system development
NES -1.36
q value < 0.05
skeletal system morphogenesis
NES -1.42
q value = 0.11
Ranked list metric
Running Enrichment Score
Position in the Ranked List of Genes
d
Col1a1
Col10a1
Sox9
Adipoq
Relative expression to β-Act
wt/wt
Δ/Δ
e
Cxcl12
Kitl
Vcam1
Angpt1
f
Oct4a
***
Nanog
Sox2
g
Adipogenic differentiation
Chondrogenic differentiation
50μm
100μm
Oil Red O
Alcian Blue

Supplementary Figure 1 POT1a deletion in MSCs leads to impaired osteo-lineage differentiation. a, *In vitro* assays with the bone marrow PDGFRα^{+} CD51^{+} MSCs isolated from Pot1a$^{flox/flox}$ / flox-stop-flox tdTomato mice. The deletion of POT1a is induced by administration of Cre recombinant vesicles *in vitro*. These images were created with BioRender.com. **b,** Pot1a flox band was deleted and Pot1a deletion band was detected in electrophoresis. **c,** Genes included in the terms “bone morphogenesis”, “ossification, skeletal system development” and “skeletal system morphogenesis” were downregulated in the POT1a deleted MSCs. **d-f,** qPCR analyses of the genes related to osteo-, adipo- and chondro- lineages (d), HSC maintenance (e), and MSC multipotency (f) in MSCs with POT1a deleted *in vitro* (n = 3 mice per group). Col1a1: Osteocyte markers, Col10a1 / Sox9: Chondrocyte markers, Adipoq: Adipocyte marker, Cxcl12 / Kitl / Vcam1 / Angpt1: HSC maintenance markers. **g,** Representative images of POT1a deleted MSCs cultured in adipo- and chondro- differentiation media, respectively. Data are represented as the mean ± SEM, ***$p < 0.001$.

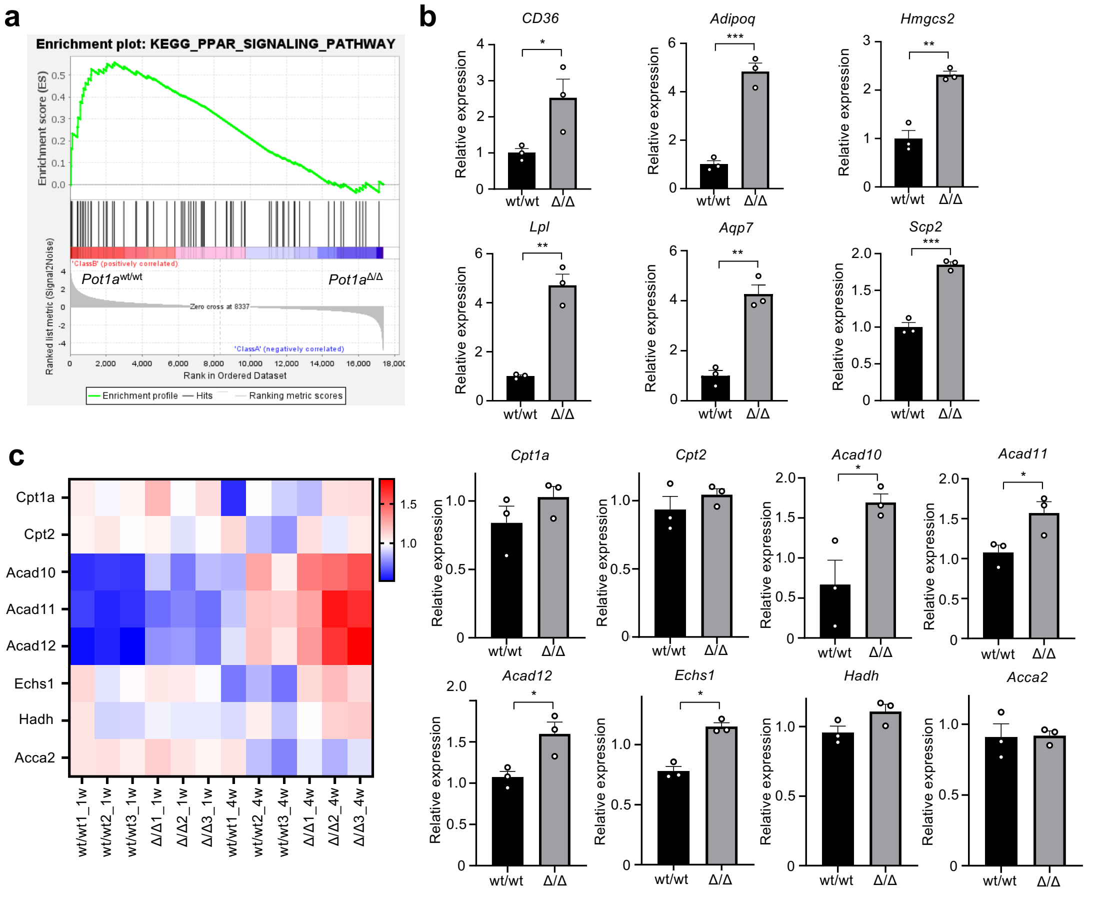

Supplementary Figure 2 POT1a deficiency induces intracellular fatty acid accumulation in MSCs. a, b, Genes associated with the term “PPAR signalling pathway” in the KEGG database were upregulated in POT1a deleted MSCs at 4 weeks. The enrichment plot (a) and expression of the genes included in the term “PPAR signalling pathway” (b). (c) Heatmap of expression of genes regulating β-oxidation of fatty acids in the KEGG database 1 and 4 weeks after deletion of POT1a. Data are represented as the mean ± SEM, *p < 0.05, **p < 0.01, ***p < 0.001.

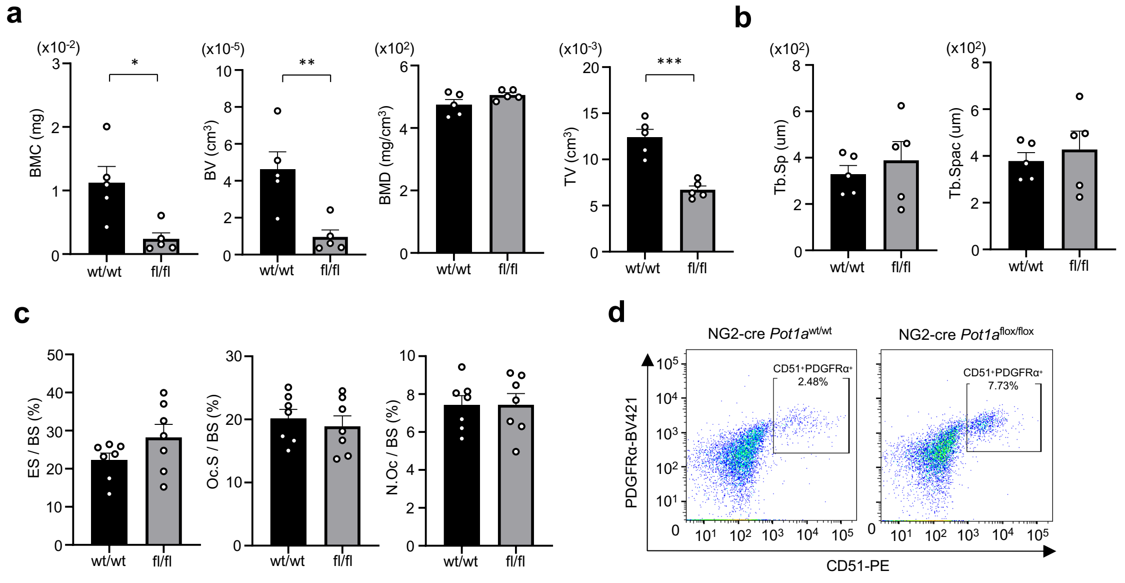

Supplementary Figure 3 MSC-targeted POT1a deficient mice exhibit impaired skeletal development. a-b, Micro CT analyses of femurs isolated from NG2-cre/Pot1a$^{fl/fl}$ mice. Comparisons of the measured parameters with controls: bone mineral content (BMC); bone volume (BV); bone mineral density (BMD); tissue volume (TV); trabecular separation (Tb.Sp) and trabecular spacing (Tb Spac) (n = 7 mice per group). **c,** Morphological analyses of the bone sections with toluidine blue staining. The measured parameters are: Eroded surface / Bone surface (ES/BS); Osteoclast surface / Bone surface (Oc.S/BS) and Osteoclast number / Bone surface (N.Oc/BS) (n = 7 mice per group). **d,** Representative FACS plots of the PDGFRα^{+} CD51^{+} MSC subpopulation in bone marrow from NG2-cre/Pot1a$^{fl/fl}$ mice. Data are expressed as the mean ± SEM, $*p < 0.05$, $**p < 0.01$, $***p < 0.001$.

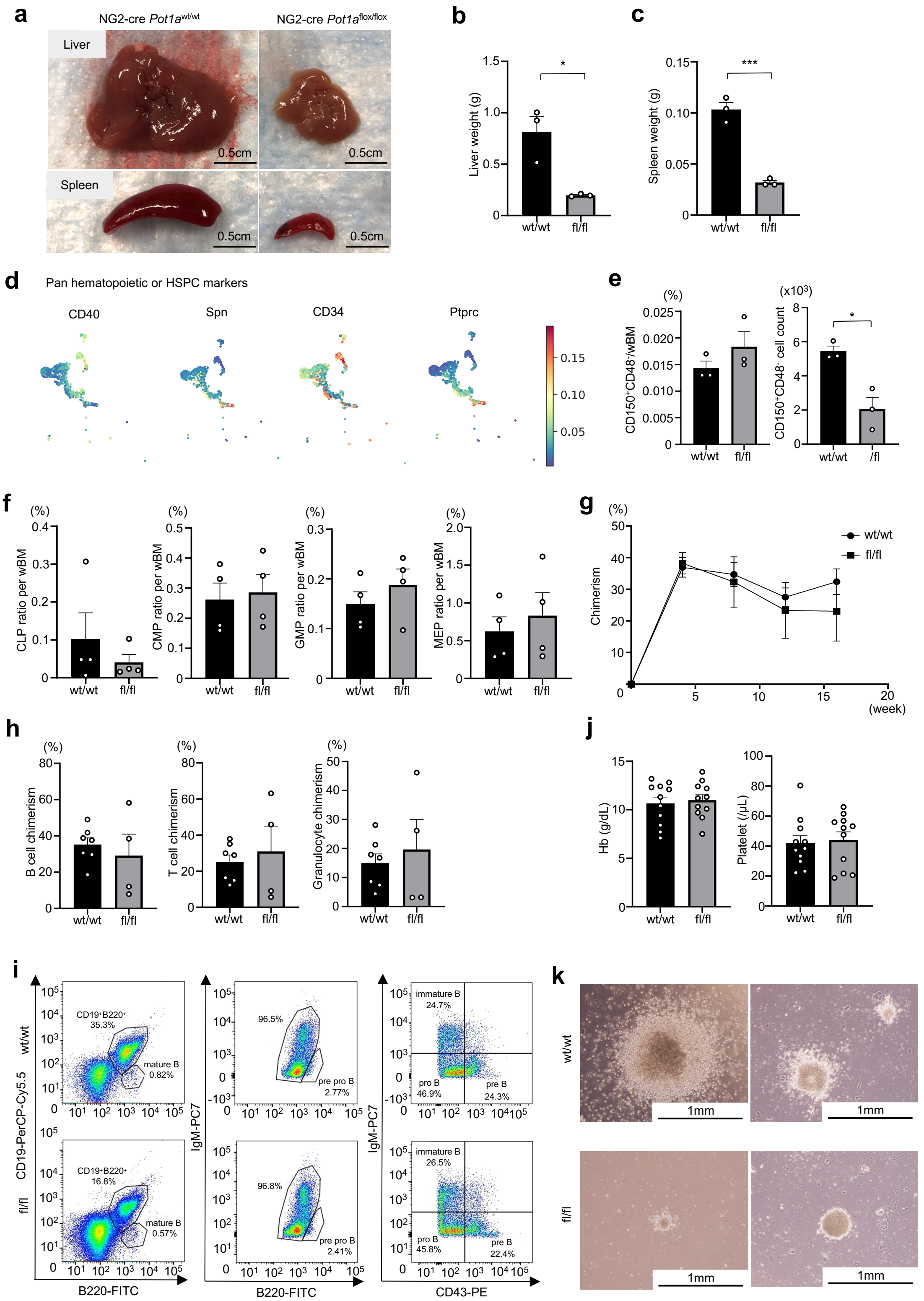

Supplementary Figure 4 Bone marrow microenvironments composed of POT1a-deleted MSCs impair B-lymphopoiesis. a-c, Liver and spleen

phenotypes of NG2-cre/Pot1a$^{fl/fl}$ mice compared to their littermates (n = 6 per mice group). **d,** Pseudotime analysis for expression heatmaps of genes related to HSPCs. **e,** Frequencies and counts of CD150^{+}CD48^{-} HSCs in whole bone marrow of NG2-cre/Pot1a$^{fl/fl}$ mice compared to their littermates (n = 4 per mice group). **f,** FACS analysis of the frequencies of more committed progenitors, such as CLP, CMP, GMP and MEP, in the NG2-cre/Pot1a$^{fl/fl}$ marrow (n = 4 per mice group). **g,** Competitive repopulation assays, in which the whole bone marrow cells from NG2-cre/Pot1a$^{fl/fl}$ or control mice were transplanted with competitor cells into lethally irradiated WT recipients. Chimerism of peripheral blood cells was analysed 1, 2, 3, and 4 months after transplant (n = 3 per mice group). **h,** Chimerism analysis of B cell, T cell and neutrophil populations 4 months after transplant. **i,** Representative FACS plots of B-cell committed progenitors (CD19^{+}B220^{+}, pre pro B, pre B, pro B, immature B, and mature B) in the NG2-cre/Pot1a$^{fl/fl}$ marrow. **j**. Measurements of haemoglobin and platelet counts (n =11 mice per group). **k**. Representative images of CFU-PreB colonies formed from the NG2-cre/Pot1a$^{fl/fl}$ marrow cells. Black arrows indicate the colonies counted as CFU-PreB. Data are expressed as the mean ± SEM, *p < 0.05, ***p < 0.001.

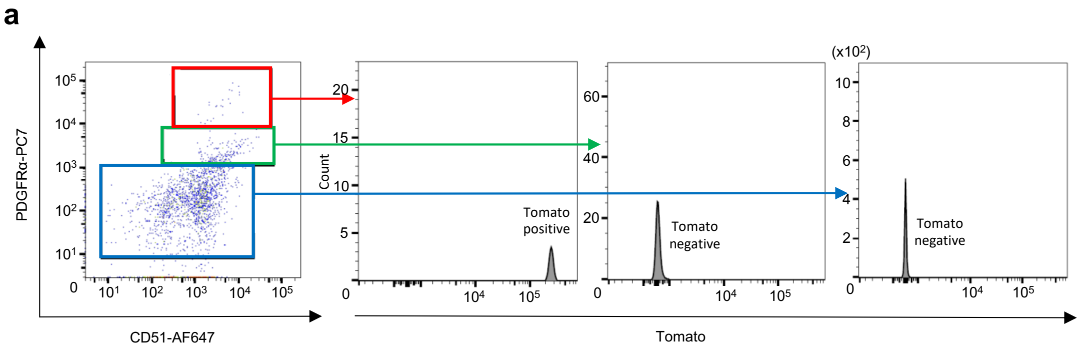

Supplementary Figure 5 POT1a deficiency in MSCs has impacts on bone growth. a, FACS analyses of bone marrow cells isolated from NG2-cre/loxp-tdTomato/Pot1a$^{fl/fl}$ mice. A dot plot and histograms show that tdTomato positive cells were exclusively found in the PDGFRα^{hi} CD51^{+} subpopulation (i.e., MSCs) within the CD45^{-} TER119^{-} CD31^{-} stromal population.

Supplementary Table 1 KEY RESOURCES TABLE

REAGENT or RESOURCE	SOURCE	IDENTIFIER
Antibodies		
BV421 anti-CD140a antibody	BD Biosciences	Cat# 562774, RRID:AB_2728781
AF647 anti-CD51 antibody	BIO-RAD	Cat# MCA2461A647T
APC/Cy7 anti-CD31 antibody	BioLegend	Cat# 102439, RRID:AB_2832288
FITC anti-CD45 antibody	BioLegend	Cat# 103108, RRID:AB_312973
PE anti-CD45.1 antibody	BD Biosciences	Cat# 553776, RRID:AB_395044
APC anti-CD45.2 antibody	BD Biosciences	Cat# 558702, RRID:AB_1645215
BV421 anti-CD45 antibody	BioLegend	Cat# 10899570, RRID:AB_103133
BV421 anti-CD31 antibody	BioLegend	Cat# 2562186, RRID:AB_102423
BV421 anti-Ter-119 antibody	BioLegend	Cat# 10933426, RRID:AB_116233
PE/Cy7 anti-Ter-119 antibody	BD Biosciences	Cat# 557853, RRID:AB_396898
PerCP-Cy5.5 anti-Ter-119 antibody	BioLegend	Cat# 116228, RRID:AB_893636
BV421 anti-c-Kit antibody	BD Biosciences	Cat# 562609, RRID:AB_11154585
AF488 anti-Sca-1 antibody	BioLegend	Cat# 122516, RRID:AB_756201
PE/Cy7 anti-Sca-1 antibody	BioLegend	Cat# 108114, RRID:AB_493596
PE anti-CD3 antibody	BioLegend	Cat# 100205, RRID:AB_312662
AF647 anti-B220 antibody	BioLegend	Cat# 103229, RRID:AB_492875
PerCP-Cy5.5 anti-CD19 antibody	BioLegend	Cat# 152406, RRID:AB_2629815
FITC anti-B220 antibody	WAKO	Cat# 1116025
PerCP-Cy5.5 anti-B220 antibody	BioLegend	Cat# 103236, RRID:AB_893354
PerCP-Cy5.5 anti-Gr-1 antibody	BD Biosciences	Cat# 560602, RRID:AB_1727563
PerCP-Cy5.5 anti-Mac-1 antibody	BioLegend	Cat# 101228, RRID:AB_893232
PE-Cy7 anti-IgM antibody	BioLegend	Cat# 406514, RRID:AB_10642031
PE anti-CD43 antibody	BioLegend	Cat# 121208, RRID:AB_493388
APC/Cy7 anti-CD48 antibody	BioLegend	Cat# 103432, RRID:AB_2561463
PE anti-CD150 antibody	BioLegend	Cat# 115903, RRID:AB_313682
AF488 anti-CD150 antibody	BioLegend	Cat# 115915, RRID:AB_528743
APC anti-Flt3 antibody	BD Biosciences	Cat# 560718, RRID:AB_1727425
PE anti-IL7Rα antibody	Tonbo Biosciences	Cat# 50-1271
PE/Cy7 anti-FcγR antibody	Tonbo Biosciences	Cat# 60-0161
Biotin anti-CD34 antibody	Thermo Fisher Scientific	Cat# 13-0341-82, RRID:AB_466425
APC streptavidin	BD Biosciences	Cat# 554067, RRID:AB_10050396
AF488 streptavidin	BioLegend	Cat# 405235
Anti-53BP1 antibody	Novus Biologicals	Cat# NB100-304
AF647 AffiniPure Donkey Anti-Rabbit IgG (H+L)	Jackson ImmunoResearch	Cat# 711-605-152
Taq Man® Assay Probes		
Pot1a	Thermo Fisher Scientific	Cat# 4331182, Mm00505816_m1
Pot1a	Thermo Fisher Scientific	Cat# 4351372, Mm01158926_m1
Actb	Thermo Fisher Scientific	Cat# 4448489, Mm00607939_s1
Gapdh	Thermo Fisher Scientific	Cat# 4448489, Mm99999915_g1
Spp1	Thermo Fisher Scientific	Cat# 4448892, Mm01204014_m1
Col1a1	Thermo Fisher Scientific	Cat# 4453320, Mm01302043_g1
Col10a1	Thermo Fisher Scientific	Cat# 4453320, Mm00487041_m1
Alpl	Thermo Fisher Scientific	Cat# 4453320, Mm00475834_m1
Pparg	Thermo Fisher Scientific	Cat# 4453320, Mm01184322_m1

Cxcl12	Thermo Fisher Scientific	Cat# 4453320, Mm00445552_m1
Vcam1	Thermo Fisher Scientific	Cat# 4453320, Mm01320970_m1
kitl	Thermo Fisher Scientific	Cat# 4453320, Mm00442972_m1
Angpt1	Thermo Fisher Scientific	Cat# 4453320, Mm00456503_m1
Cxcl12	Thermo Fisher Scientific	Cat# 4453320, Mm00445552_m1
IL-7	Thermo Fisher Scientific	Cat# 4453320, Mm01295803_m1
IL-7	Thermo Fisher Scientific	Cat# 4448892, Mm01295804_m1
IL-7	Thermo Fisher Scientific	Cat# 4448892, Mm01295805_m1
IL-7	Thermo Fisher Scientific	Cat# 4453320, Mm00434291_m1
Chemicals, Peptides, and Recombinant Proteins		
Cre Recombinase Gesicles	Takara	Cat# 631449
RPMI 1640	SIGMA	Cat# R8758
MEM Alpha (1X)	Thermo Fisher Scientific	Cat# 12571-063
Penicillin Streptomycin	Thermo Fisher Scientific	Cat# 15140122
Collagenase Type 1	Wothington Biochemical	Cat# LS004196
Collagenase IV	Thermo Fisher Scientific	Cat# 17104019
Dispase	Thermo Fisher Scientific	Cat# 17105041
MesenCult expansion kit	STEMCELL technologies	Cat# ST_05513
Mouse Mesenchymal Stem Cell Functional Identification Kit	R&D SYSTEMS	Cat# SC010
Absolute Human Telomere Length Quantification qPCR Assay Kit	ScienCell	Cat# 8918
FCCP	Merck (Sigma-Aldrich)	Cat# C2920
rotenone	Merck (Sigma-Aldrich)	Cat# R8875
antimycin	Merck (Sigma-Aldrich)	Cat# A8674
oligomycin	Merck (Sigma-Aldrich)	Cat# O4876
etomoxir	Merck (Sigma-Aldrich)	Cat# E1905
saponin	Merck (Sigma-Aldrich)	Cat# SAE0073
CellROX™ Deep Red Reagent	Thermo Fisher Scientific	Cat# C10422
MitoTracker Deep Red FM	Thermo Fisher Scientific	Cat# M22426
BODIPY 493/503	Thermo Fisher Scientific	Cat# D3922
Experimental Models: Organisms/Strains		
C57BL/6 mice (B6-Ly5.1)	Japan Clea	N/A
C57BL/6 mice (B6-Ly5.2)	Sankyo Laboratory	N/A
NG2-Cre mice (B6.FVB-Tg(Cspg4-cre)1RkI/J)	Jackson Laboratory	N/A
tdTomato-flox mice (B6.Cg-Gt(ROSA)26Sor$^{tm9(CAG-tdTomato)Hze}$/J)	Jackson Laboratory	N/A
Pot1a-flox mice (B6;129-Pot1a$^{tm1.1Tdl}$/J)	Jackson Laboratory	N/A
Software and Algorithms		
BD FACSDiva Software v8.0	BD	N/A
FlowJo software	BD	N/A
GraphPad Prism8	MDF	N/A
Others		
CD45 MicroBeads	Miltenyi Biotec	Cat# 130-052-301
Ter-119 MicroBeads	Miltenyi Biotec	Cat# 130-049-901
