# Supplementary material for: POT1a deficiency in mesenchymal niches perturbs B-lymphopoiesis
Source: Commun Biol. 2023 Sep 29;6:996. doi: 10.1038/s42003-023-05374-0 (PMC10541440; doi:10.1038/s42003-023-05374-0)
Supplement: Supplementary file 3 — Description of Additional Supplementary Data [file 42003_2023_5374_MOESM3_ESM.docx]

**Description of Additional Supplementary Files**

**File name:** Supplementary Data 1

**Description:** The exact source data for all the graphs and charts in the figure 1-5.
