# Supplementary material for: POT1a deficiency in mesenchymal niches perturbs B-lymphopoiesis
Source: Commun Biol. 2023 Sep 29;6:996. doi: 10.1038/s42003-023-05374-0 (PMC10541440; doi:10.1038/s42003-023-05374-0)
Supplement: Supplementary file 5 — Reporting Summary [file 42003_2023_5374_MOESM5_ESM.pdf]

nature portfolio

Corresponding author(s): Fumio Arai (COMMSBIO-22-1294-T)

Last updated by author(s): Aug 27, 2023

Reporting Summary

Nature Portfolio wishes to improve the reproducibility of the work that we publish. This form provides structure for consistency and transparency in reporting. For further information on Nature Portfolio policies, see our Editorial Policies and the Editorial Policy Checklist.

Statistics

For all statistical analyses, confirm that the following items are present in the figure legend, table legend, main text, or Methods section.

n/a	Confirmed	
☐	☒	The exact sample size (*n*) for each experimental group/condition, given as a discrete number and unit of measurement
☐	☒	A statement on whether measurements were taken from distinct samples or whether the same sample was measured repeatedly
☐	☒	The statistical test(s) used AND whether they are one- or two-sided<br>*Only common tests should be described solely by name; describe more complex techniques in the Methods section.*
☒	☐	A description of all covariates tested
☒	☐	A description of any assumptions or corrections, such as tests of normality and adjustment for multiple comparisons
☒	☐	A full description of the statistical parameters including central tendency (e.g. means) or other basic estimates (e.g. regression coefficient) AND variation (e.g. standard deviation) or associated estimates of uncertainty (e.g. confidence intervals)
☒	☐	For null hypothesis testing, the test statistic (e.g. *F*, *t*, *r*) with confidence intervals, effect sizes, degrees of freedom and *P* value noted<br>*Give P values as exact values whenever suitable.*
☒	☐	For Bayesian analysis, information on the choice of priors and Markov chain Monte Carlo settings
☒	☐	For hierarchical and complex designs, identification of the appropriate level for tests and full reporting of outcomes
☒	☐	Estimates of effect sizes (e.g. Cohen's *d*, Pearson's *r*), indicating how they were calculated

Our web collection on statistics for biologists contains articles on many of the points above.

Software and code

Policy information about availability of computer code

Data collection	FACS data were analyzed with FlowJo software (BD Biosciences).
Data analysis	Statistical analyses were performed with GraphPad Prism 8 (version 8.3.0) (MDF). Q-PCR data were analyzed by CFX Manager Software v2.1. Three-dimensional microstructural image data of micro CT were reconstructed and structural indices were calculated using a TRI/3D-BON software (Ratoc System Engineering). Bone images were acquired by a light microscope (Axio Imager 2; Zeiss) and all histological analyses performed using a WinROOF 2013 software (Mitani).

For manuscripts utilizing custom algorithms or software that are central to the research but not yet described in published literature, software must be made available to editors and reviewers. We strongly encourage code deposition in a community repository (e.g. GitHub). See the Nature Portfolio guidelines for submitting code & software for further information.

Data

Policy information about availability of data

All manuscripts must include a data availability statement. This statement should provide the following information, where applicable:

- Accession codes, unique identifiers, or web links for publicly available datasets
- A description of any restrictions on data availability
- For clinical datasets or third party data, please ensure that the statement adheres to our policy

RNA sequence data are available at GEO under accession number GSE197720 and GSE197721.

Field-specific reporting

Please select the one below that is the best fit for your research. If you are not sure, read the appropriate sections before making your selection.

☒ Life sciences ☐ Behavioural & social sciences ☐ Ecological, evolutionary & environmental sciences

For a reference copy of the document with all sections, see nature.com/documents/nr-reporting-summary-flat.pdf

Life sciences study design

All studies must disclose on these points even when the disclosure is negative.

Sample size	No statistical method was used to pre-determine sample size. Sample sizes were chosen according to commonly used standards in the field. The number of independent experiments are indicated in the legend of each Figure.
Data exclusions	No data were excluded from experiments presented in this manuscript.
Replication	All experiments obtained results that were reproduced typically on a minimum of three independently obtained samples.
Randomization	Cells used for imaging were selected randomly and analyzed equally with no sub-sampling and thus, there was no requirement for randomization.
Blinding	For all experiments, either the differences between samples were obvious making blinding impossible or blinding was not applicable due to the nature of the experiment.

Reporting for specific materials, systems and methods

We require information from authors about some types of materials, experimental systems and methods used in many studies. Here, indicate whether each material, system or method listed is relevant to your study. If you are not sure if a list item applies to your research, read the appropriate section before selecting a response.

Materials & experimental systems

n/a	Involved in the study	
☐	☒	Antibodies
☒	☐	Eukaryotic cell lines
☒	☐	Palaeontology and archaeology
☐	☒	Animals and other organisms
☒	☐	Human research participants
☒	☐	Clinical data
☒	☐	Dual use research of concern

Methods

n/a	Involved in the study	
☒	☐	ChIP-seq
☐	☒	Flow cytometry
☒	☐	MRI-based neuroimaging

Antibodies

Antibodies used	The following monoclonal antibodies (Abs) were used for flow cytometry and cell sorting: anti-c-Kit (2B8, BD Biosciences), -Sca-1 (E13-161.7 / D7, BioLegend), -CD140a (APA5, BD Biosciences), -CD51 (RMV-7, BIO-RAD), -CD4 (RM4-5, BioLegend), -CD8a (53-6.7, BioLegend), -B220 (RA3-6B2, BioLegend / WAKO), -CD31 (390, BioLegend), -TER-119 (TER-119, BioLegend / BD Biosciences), -Gr-1 (RB6-8C5, BD Biosciences), -Mac-1 (M1/70, BioLegend), -CD48 (HM48-1, BD Biosciences), -CD150 (TC15-12F12.2, BioLegend), -CD19 (6D5, BioLegend), -IgM (MHM-88, BioLegend), -CD43 (S11, BioLegend), -CD45.1 (A20, BD Biosciences), -CD45.2 (104, BD Biosciences), -CD45 (30-F11, BioLegend), -Flt3 (A2F10.1, BD Biosciences), -IL7Rα (A7R34, TONBO biosciences), -FcγR (2.4G2, TONBO biosciences), -53BP1 (Novus Biologicals). A biotinylated CD34 (RAM34, Thermo Fisher Scientific) and a fluorochrome labeled streptavidin (BD Biosciences / BioLegend) was used.
Validation	Antibodies were all validated by commercial source. No home-made or previously unpublished antibodies were used in these studies.

Animals and other organisms

Policy information about studies involving animals; ARRIVE guidelines recommended for reporting animal research

Laboratory animals	C57BL/6 (B6-Ly5.2) were purchased from CLEA Japan, Inc.. Congenic C57BL/6 mice for the Ly5 locus (B6-Ly5.1) were purchased from Sankyo lab. POT1afl/fl (B6;129-Pot1atm1.1Tdl/J), NG2-cre (B6.FVB-Tg(Cspg4-cre)1Rkl/J) and flox-stop-flox tdTomato (B6.Cg-Gt(ROSA)26Sortm9(CAG-tdTomato)Hze/J) mice were provided by the Jackson laboratory.
Wild animals	This study did not involve wild animals.

Field-collected samples | This study did not involve samples collected from the field.

Ethics oversight | All experiments were carried out in accordance with ARRIVE Guideline and the Guidelines for Animal and Recombinant DNA experiments at Kyushu University.

Note that full information on the approval of the study protocol must also be provided in the manuscript.

Flow Cytometry

Plots

Confirm that:

☒ The axis labels state the marker and fluorochrome used (e.g. CD4-FITC).

☒ The axis scales are clearly visible. Include numbers along axes only for bottom left plot of group (a 'group' is an analysis of identical markers).

☒ All plots are contour plots with outliers or pseudocolor plots.

☒ A numerical value for number of cells or percentage (with statistics) is provided.

Methodology

Sample preparation	Samples were obtained and treated as described in methods section.
Instrument	Stained cells were analyzed and sorted using a FACS AriaIII (BD Biosciences). Data were analyzed with FlowJo software (BD Biosciences).
Software	Flow cytometry analysis was performed with a FACS Verse using FACSuite software (BD Biosciences).
Cell population abundance	Pure population of cells were analyzed and sorted by FACS.
Gating strategy	Cells were gated using Forward Scatter Area (FSC-A) vs Side Scatter Area (SSC-A) to remove debris. Cell populations were gated on live cells and defined as: MSCs: CD45-CD31-Ter119-PDGFRA+CD51+, pre pro B: CD19+B220+IgM-, pre B: CD19 +B220-CD43+IgM-, pro B: CD19+B220-CD43-IgM-, immature B: CD19+B220-CD43-IgM+, mature B: CD19-B220+.<br>The other gating strategies are indicated in the legend of each Figure.

☒ Tick this box to confirm that a figure exemplifying the gating strategy is provided in the Supplementary Information.
